# Core Microbial Functional Activities in Ocean Environments Revealed by Global Metagenomic Profiling Analyses

**DOI:** 10.1371/journal.pone.0097338

**Published:** 2014-06-12

**Authors:** Ari J. S. Ferreira, Rania Siam, João C. Setubal, Ahmed Moustafa, Ahmed Sayed, Felipe S. Chambergo, Adam S. Dawe, Mohamed A. Ghazy, Hazem Sharaf, Amged Ouf, Intikhab Alam, Alyaa M. Abdel-Haleem, Heikki Lehvaslaiho, Eman Ramadan, André Antunes, Ulrich Stingl, John A. C. Archer, Boris R. Jankovic, Mitchell Sogin, Vladimir B. Bajic, Hamza El-Dorry

**Affiliations:** 1 Department of Biology and the Science and Technology Research Center, School of Sciences and Engineering, The American University in Cairo, Cairo, New Cairo, Egypt; 2 Departamento de Bioquímica, Instituto de Química, Universidade de São Paulo, São Paulo, Brazil; 3 Escola de Artes, Ciências e Humanidades, Universidade de São Paulo, São Paulo, Brazil; 4 Computational Bioscience Research Center, King Abdullah University of Science and Technology, Thuwal, Kingdom of Saudi Arabia; 5 Institute for Biotechnology and Bioengineering, Centre of Biological Engineering, University of Minho, Portugal; 6 Red Sea Research Center, King Abdullah University of Science and Technology, Thuwal, Kingdom of Saudi Arabia; 7 Josephine Bay Paul Center, Marine Biological Laboratory, Woods Hole, Massachusetts, United States of America; Universidade Federal do Rio de Janeiro, Brazil

## Abstract

Metagenomics-based functional profiling analysis is an effective means of gaining deeper insight into the composition of marine microbial populations and developing a better understanding of the interplay between the functional genome content of microbial communities and abiotic factors. Here we present a comprehensive analysis of 24 datasets covering surface and depth-related environments at 11 sites around the world's oceans. The complete datasets comprises approximately 12 million sequences, totaling 5,358 Mb. Based on profiling patterns of Clusters of Orthologous Groups (COGs) of proteins, a core set of reference photic and aphotic depth-related COGs, and a collection of COGs that are associated with extreme oxygen limitation were defined. Their inferred functions were utilized as indicators to characterize the distribution of light- and oxygen-related biological activities in marine environments. The results reveal that, while light level in the water column is a major determinant of phenotypic adaptation in marine microorganisms, oxygen concentration in the aphotic zone has a significant impact only in extremely hypoxic waters. Phylogenetic profiling of the reference photic/aphotic gene sets revealed a greater variety of source organisms in the aphotic zone, although the majority of individual photic and aphotic depth-related COGs are assigned to the same taxa across the different sites. This increase in phylogenetic and functional diversity of the core aphotic related COGs most probably reflects selection for the utilization of a broad range of alternate energy sources in the absence of light.

## Introduction

Microbial communities dominate the oceans and seas [1] and are involved in fundamental processes in the global ecosystem, synthesizing half of the photosynthetic biomass and producing significant amounts of oxygen [2]. They also play pivotal roles in carbon, nitrogen, sulfur and phosphorus cycles [3], [4], [5], [6]. The diverse capabilities of marine microbes are the result of selection and adaptation to environments in which light level, oxygen concentration, nutrient availability, salinity, temperature and hydrostatic pressure all vary widely [7], [8], [9], [10], [11], [12], [13], [14], [15], [16], [17].

Acquisition and loss of genetic information, and mutational variation of gene regulatory networks, are known to shape the lifestyles of microorganisms including those that reside in marine environments [18], [19], [20], [21], [22], [23], [24], [25]. However, some basic questions regarding these processes in marine environments remain unresolved. What metabolic and physiological capabilities enable microbes to thrive in marine habitats? How do biotic and abiotic factors, including human activities and climate change, affect their capacity to adapt to a specific environment? Thanks to recent developments in molecular biology, remote sensing and deep-sea sampling, these issues can now be addressed [26], [27], [28].

Metagenomic sampling of marine microbial communities at various sites has contributed much to our understanding of their biochemical and physiological diversity [7], [8], [9], [10], [11], [12], [13], [14], [29], [30], [31]. However, a comprehensive comparative analysis of publicly accessible sequences from a wide range of depth-related locations in oceans, which is necessary for a better understanding of the distribution and dynamics of microorganisms in marine ecosystems, has been lacking. In this study we report such an analysis, based on 24 metagenomic datasets from known depths at 11 sites representing a wide range of marine settings. These comprise all publicly available data from studies that generated sufficient sequence coverage to permit broad comparative analysis.

Four of the datasets come from samples collected at different depths in the Atlantis II Basin in the Central Red Sea (ATII), and are reported here for the first time. In addition, we included in our analysis three collections of datasets, each representing four different depths in the water column, from ALOHA Station in the North Pacific Subtropical Gyre [32], BATS Station in the Sargasso Sea [32], and Station 3 off the Northern Chilean coast near Iquique (Iquique) [31]; four datasets derived from samples obtained at depths of 1,000 m in the Central Basin of the Sea of Marmara [8], 4,000 m at ALOHA [9], 6,000 m within the Puerto Rico Trench (PRT) [11], and 50 m in the Mediterranean off Alicante in Southeastern Spain (Med.) [10]. We also selected for inclusion in our global study just four sites from the vast array of near-surface datasets acquired by the Global Ocean Sampling (GOS) expedition [7],[13]. The entire collection comprises approximately 12.0 million sequences encompassing 5,358 Mb.

As a benchmark for understanding the genomic changes underlying microbial adaptation to different light and oxygen regimes, we assembled a core set of reference depth-related photic and aphotic COGs and defined a group of functional activities associated with extreme hypoxia, based on functional profiling of all 24 datasets mentioned above. These were then utilized to characterize biological activities in two specific oceanographic settings in which the normal relationships between light, oxygen and depth are disrupted: the Humboldt Current System (HCS) - a major upwelling zone - and the salinity-based two-layer flow system in the Sea of Marmara. Phylogenetic assignment of core COGs indicated that the same microbial taxa dominated the photic zone across all the sites considered, while aphotic zones show greater diversification and differentiation.

## Materials and Methods

### Sample collection, DNA isolation, and Whole-genome shotgun pyrosequencing

Water column samples from Atlantis II Basin in the Red Sea (21°13’ N, 37°58’ E) were obtained during Leg2 of the WHOI/KAUST/HCMR sampling cruise aboard the R/V Aegeo in April 2010. Permits to collect water samples from all the depths of Atlantis II basin in the Red Sea were issued by the Ministry of Defense, Kingdome of Saudi Arabia. All the field studies did not involve endangered or protected species. Detailed of samples collection and processing, environmental DNA isolation, whole-genome shotgun pyrosequencing, and establishing of ATII depth-related datasets for the four depths are presented in Methods S1.

### Nucleotide sequence accession numbers

The ATII 454 metagenome has been deposited in the GenBank Sequence Read Archive with the following accession numbers: ATII 50 m: SRS598124; ATII 200 m: SRS598125; ATII 700 m: SRS598128; and ATII 1500 m: SRS598129.

### ATII and publicly available datasets employed in comparative analysis

In addition to the four ATII depth-related datasets newly generated for this work, metagenomic datasets from ten other sites were obtained from public databases (fig. S1 and table S1 in file S1). Detailed information regarding the publically available datasets, 454 shotgun read simulation, sub-sampling, functional assignment using the eggNOG database, and analysis of differentially abundant COGs are presented in Methods S1.

### Comparative analysis of datasets based on COG abundance

Because there were three depth-related metagenomic collections of datasets that could be used as reference (ALOHA, BATS and ATII), we elected to first screen for COGs that differ significantly in abundance among the various depths in each of the three water columns. This was achieved by means of Fisher's exact test (p≤0.01, FDR-corrected), using the exact Test function of the R bioconductor package edgeR [33], in order to identify common COGs that displayed significant differences in normalized abundance levels between at least two depths in one of the three water columns. 176 depth-related COGs were identified at this step and were employed for hierarchical clustering of all datasets (table S2 in file S1).

### Determination of reference photic/aphotic global-core depth-related COGs

Datasets were initially classified as photic or aphotic based on publicly available PAR and oceanographic data. Subsequently the average normalized abundances of each COG were determined for the identified photic and aphotic groups of datasets, obtained by the hierarchical clustering of the 24 datasets based on the normalized abundances of the 176 depth-related COGs previously identified (table S3 in file S1).

Then, unequal variance conservative Student's t-test (Welch's test; p≤0.05, FDR-corrected) was employed to determine the COGs that presented statistically significant differences in the means of the two groups (n = 12 for each group). 82 COGs were found to vary significantly (p≤0.0001, FDR-corrected) in abundance between the photic and aphotic datasets and considered subsequently as the reference photic/aphotic global-core depth-related set of COGs. Out of those, 54 COGs were assigned as photic and 28 COGs were assigned as aphotic based on the means of the normalized abundance of each COGs between the photic and aphotic datasets (tables S4A and S4B in file S1).

### Comparison of photic to aphotic depth-related COG abundance ratios in water columns

Using the reference photic/aphotic global-core depth-related set of COGs, the normalized abundance means of the reference 54 photic and 28 aphotic depth-related COGs were determined for each dataset. The ratio of photic to aphotic normalized abundance means of each dataset was then calculated and log2 transformed in order to be plotted. The average and mean standard deviations of the log2-transformed ratios were determined for photic and aphotic datasets from ATII, ALOHA and BATS to establish ratio ranges indicative of photic or aphotic genomic content.

### Assessment of the effect of hypoxic environment on COG abundance

To evaluate the effect of low oxygen concentration on the genomic content of microbial communities, COGs that presented statistically significant changes in normalized abundance levels between datasets of the Iquique water column were initially identified following the same procedure performed for the selection of differentially abundant depth-related COGs in the three reference columns (ATII, ALOHA, BATS). Such analysis resulted in a list of 162 COGs from which the 82 global photic/aphotic common-core depth-related COGs were subsequently excluded to avoid influence of sunlight intensity level on the identification of abundance changes specifically related to dissolved oxygen concentration (D.O.). Datasets were initially divided into two groups based on publicly available dissolved oxygen concentration (D.O.) data for the 24 datasets analyzed. Iquique datasets from 85 m, 110 m and 200 m depths (D.O.<20 µmol.kg-1) were considered as the low-oxygen standard (n = 3) to which all other remaining datasets (n = 21) were compared. Fisher's exact test was again employed to determine the COGs that were statistically different in abundance across the Iquique water column. COGs whose abundances had been shown to vary in association with light level were omitted from this analysis. Wilcoxon's test was then used to find the COGs whose normalized abundances varied in association with oxygen concentration. 18 COGs (table S5 in file S1) were found to vary significantly (p≤0.05, FDR-corrected) in abundance between low and high oxygen datasets.

### Taxonomic distribution of photic/aphotic depth-related COGs among sites

The taxonomic distribution analysis was performed on selected datasets representing euphotic and aphotic zones of the different oceanic sites sampled. Datasets obtained from the shallowest samples of the reference water columns (ATII, ALOHA and BATS) plus the four GOS and the Mediterranean ones were used as representative of the euphotic zone, whilst datasets from the deepest samples of the reference water columns and those from Marmara, PRT and ALOHA (4,000 m) corresponded to the aphotic zone in the analysis. Reads from each dataset mapped to the photic/aphotic core related COGs were subjected to BLASTX similarity search against the NCBI nr database using a maximum e-value threshold of 1e-05 [34]. A taxonoxomic identifier was assigned to each individual read based on the best BLAST hit using NCBI taxdump (version of March, 2013). Taxonomic counts were then summarized at the phylum level per COG for each analyzed dataset (tables S6A and S6B in file S1) by means of bash/perl scripts developed in-house. Eukarya or unidentified taxa were discarded from the analysis. Heatmaps were generated from the summarized raw counts for the core photic and aphotic depth-related COGs using the publicly available software environment R version 2.11.1 (www.r-project.org, RColorBrewer and gplots libraries).

## Results and Discussion

### Defining the reference depth-related environments

The metagenomic datasets employed in this work were derived from samples obtained at different sites in the world's oceans. Detailed information on sites and sampling methods is presented in figure S1, and table S1 in file S1. In order to avoid biases in the comparison of samples due to different sequencing methods and coverage, simulated 454 sequences were generated from datasets made up of assembled contigs (ALOHA 4,000 and GOS datasets) prior to extraction from all analyzed datasets of random subsamples of the same size as the smallest complete collection (BATS 500 m; for details, see Methods S1).

**Figure 1:**
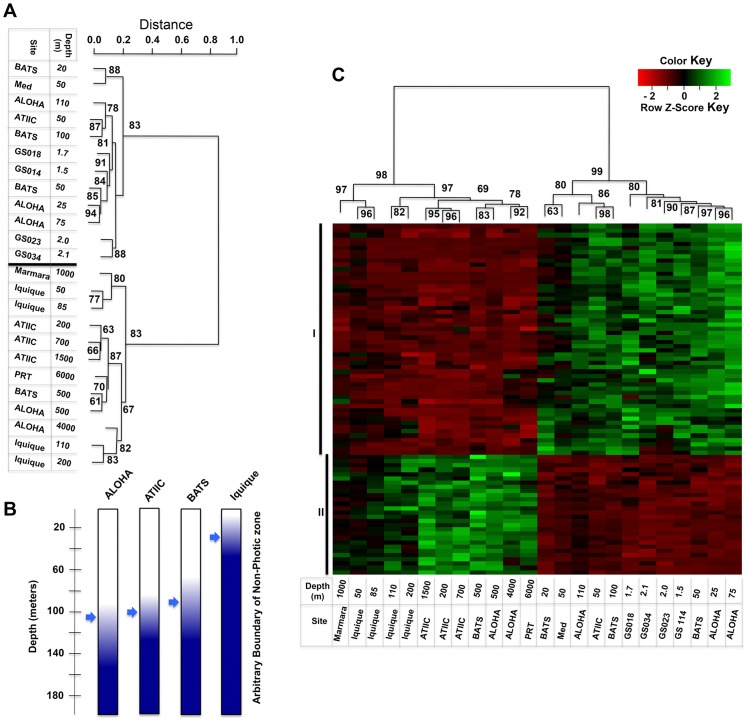
Clustering of the 176 depth-related COGs. (**A**) Hierarchical clustering of the 176 depth-related COGs in the 24 datasets. Clustering analysis is based on the normalized abundance profile of the 176 depth-related COGs that were shared by the three reference water columns (ATII, ALOHA and BATS) and significantly differed in abundance within at least one of them (details in Materials and Methods). The height indicates the relative distance between datasets. Bootstrap confidence values above 60 for the nodes are shown. The heatmap is shown in fig. S3A. (**B**) Location of the boundary between the photic and aphotic zones in each of the four water columns. The arrows indicate the depth at which PAR reaches 1% of the level at the surface. (**C**) Hierarchical clustering of the photic/aphotic global-core depth-related COGs. 82 COGs that showed statistically significant difference (Welch's test, FDR-corrected, p≤1E-04) in their normalized abundance between the photic and the aphotic groups of datasets were selected to establish a photic/aphotic global-core, depth-related reference set. 54 COGs had significantly higher abundance in the photic datasets (Group I, table S4A in file S1); contrary to the remaining 28 aphotic related COGs (Group II, table S4B in file S1). Bootstrap confidence values for the major nodes are shown. Heatmap coloring reflects the Z score of normalized abundances of each COG across all clustered datasets (details in Materials and Methods).

It is important to emphasize that the datasets analyzed here include all those based on sampling at multiple depths in water columns at single sites: ALOHA [32], BATS [32], Iquique [31], and ATII (this work). The Iquique site [31] lies within the Humboldt Current System (HCS), the most productive marine ecosystem in the global oceans [35], which dramatically affects environmental conditions at this location [35]. The HC flows along the West coast of South America and drives a major upwelling system that brings cold, nutrient-rich water toward the surface of the Eastern Tropical South Pacific off Northern Chile. This promotes the growth of a dense planktonic community and supports a high level of primary production [35]. Among the most notable effects is the efficient depletion of dissolved oxygen, from >200 μmol.kg^−1^ at the surface to 3 to 4 μmol.kg^−1^ at depths between 110 and 200 m, by aerobic heterotrophs [31], [36]. In addition, due to dense growth of phytoplankton and the presence of organic particulates, the sunlit zone at the Iquique site is rather shallow compared to the other sites (fig. 1B). We therefore considered the water columns at ALOHA, BATS and Atlantis II, but not that at Iquique, as reference depth-related environments.

### Clustering COGs according to depth

To identify known biochemical functions represented in the 24 datasets, we subjected each one to sequence similarity analysis using the eggNOG v2.0 database [37], and calculated the normalized abundances of the identified clusters of orthologous groups of proteins (COGs) [38]. COGs that exhibited statistically significant differences in abundance within the three reference water columns were retrieved individually using Fisher's exact test (p ≤0.01, and FDR-corrected; for details see Methods S1). This resulted in 649, 413, and 463 COGs for ATII, ALOHA and BATS, respectively. Of these, 176 COGs were present at all three sites (table S2 in file S1).

We first performed hierarchical clustering on the normalized abundance data for these 176 COGs in the three reference columns. The major feature common to the ATII, ALOHA and BATS profiles is the division of the datasets into two depth-related groups, representing the surface/near-surface zone, and a deeper zone (fig. S2, and table S2 in file S1).

Next, we proceeded to a hierarchical clustering of the same 176-COG set in all 24 datasets. As expected for a generalized depth-related pattern, members of the set represented in the Sea of Marmara (1,000 m), ALOHA (4,000 m) and the Puerto Rico Trench (PRT, 6,000 m) samples clustered with the deeper water group, while those occurring in the four GOS surface-water and the 50 m Mediterranean Sea datasets fell into the surface/near-surface group (fig. 1A, and table S3 in file S1). This depth-related division reflects metabolic and physiological processes specific to the microbial communities residing in photic and aphotic zones. Note that the dividing line coincides with the bottom of the photic zones in the three columns, as determined by their PAR (photosynthetically active radiation) values (fig. 1B). Interestingly, inclusion of the Iquique datasets in the clustering analysis preserves the depth-related profile division found at the other ten sites. However, as shown in fig. 1A, all four datasets generated from the Iquique samples (50 m, 85 m, 110 m and 200 m) clustered with the aphotic group. Nevertheless, the apparently anomalous position of the datasets related to the Iquique column in the profile of the 11 sites (fig. 1A) is entirely compatible with the environmental conditions at the site [31] which, as mentioned above, differ quite significantly from those at ALOHA [12], BATS [39] and ATII sites. The PAR value at Iquique drops below 1% of its surface value at a depth of less than 50 m [31] (fig. 1B). If one defines this level as the lower limit of the photic zone, it is not surprising that all Iquique datasets cluster with the aphotic group.

### The photic/aphotic global-core depth-related COGs

In order to identify specific photic/aphotic global-core depth-related COGs we compared the normalized abundance of the photic with the aphotic groups of datasets defined in the dendrogram shown in fig. 1A. Of the 176 COGs used in the construction of this profile, a major subset of 82 COGs (19%) were found to show a statistically significant difference in abundance between photic and aphotic datasets (p≤1e-04 and FDR corrected; see details in Materials and Methods). Based on the mean normalized abundance for the whole set, 54 were considered photic related COGs and 28 were regarded as aphotic related COGs (fig. 1C, see also tables S4A and S4B in file S1). Bootstrapping evaluation indicated a high degree of confidence in the division of the photic and aphotic groups of the datasets presented in fig. 1A and 1C. This reflects the fact that functional adaptation to ambient light level is most probably one of the factors that strongly constrains the genetic variability of microorganisms in ocean environments. Thus, COGs related to photosynthesis, biosynthesis of light-harvesting pigments, assimilation of carbon dioxide by photosynthetic bacteria, as well as light-induced DNA repair and oxidative stress responses, were markedly more abundant in the photic than the aphotic datasets, as were COGs related to nitrogen fixation and phosphate metabolism. Conversely, in the aphotic datasets, COGs related to protein and amino acid catabolism, methane oxidation, sulfate assimilation and metabolism, selenocysteine metabolism and terpenoid biosynthesis were overrepresented, mirroring the exploitation of other energy sources in this zone.

It is important to note that previously detected differences in microbial gene abundances between the surface zone and the deep sea have been based on comparisons of limited metagenomic datasets [9], [11], [12]. In the present work all sufficiently extensive depth-related datasets available from different sites around the world's oceans (fig. S1, and table S1 in file S1) have been analyzed in a uniform fashion, allowing us to establish clearly defined sets of depth-related biological functions for the oceans as a whole. Such core functions shared between distinct sites around the global ocean can thus be used as a reliable diagnostic tool to analyze the distribution of light-related biological activities in sites that present atypical environmental conditions, as described below.

### Abundance ratio of photic to aphotic depth-related COGs in the major upwelling zone of the Humboldt Current System and the salinity-based two-layer flow system in the Sea of Marmara

**Figure 2:**
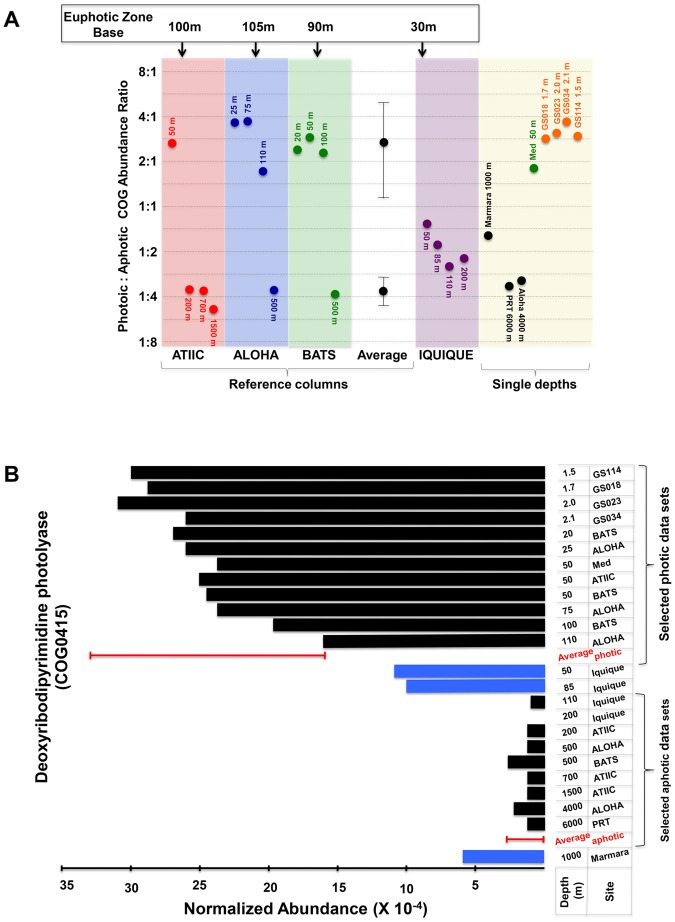
Abundance ratios of photic to aphotic related COGs. (**A**) Mean abundance ratios of photic to aphotic related COGs for datasets from the reference water columns, the Iquique water column, and from single-depth datasets. This analysis was initially determined using the 82-COG global-core, depth-related reference set, as described in Materials and Methods. Applying the ratios for the photic and aphotic datasets from the reference water columns, ratio ranges (mean±two SD) were established to indicate photic or aphotic functional genomic content enrichment. Mean abundance ratios for the Iquique water column and the single-depth datasets were calculated to verify their balance between photic and aphotic related biological functions. The arrows at the top of the diagram indicate the approximate depth of the boundary between photic and aphotic zones in each water column (fig. 1B). (**B**) Normalized abundance of the deoxyribodipyrimidine photolyase gene (COG0415; table S4A in file S1) in the 24 datasets obtained from the indicated depths. The average normalized abundance was calculated for the photic and for the aphotic groups of datasets. The bars represent two SD of the mean.

The environmental differences between the water columns at ALOHA, BATS and ATII relative to Iquique provide an excellent opportunity to explore the interplay between environmental factors - such as nutrient availability, light intensity, oxygen concentration - and the genomic changes that underlie microbial adaptation. When we examined the abundance ratio of the photic to the aphotic related COGs in all four water columns, we found, as expected, a well-defined correlation with the photic/aphotic boundary in the cases of ALOHA, BATS and ATII (fig. 2A) – high ratios of photic to aphotic related COG abundance in the photic samples and low ratios in aphotic ones. However, in the Iquique column the aphotic:photic ratio is merely 1.3 for the 50 m sample and 1.8 for the 85 m sample, whilst the mean ratio for the aphotic datasets of the reference columns is 3.8 (fig. 2A). The fact that microbial communities in this zone are genetically equipped for photosynthesis strongly suggests that the 50 m and 85 m levels are part of the mixed-water layer at this site.

To investigate this issue further, we inspected the ratios of photic to aphotic related COGs in all 24 datasets in relation to light levels. We selected one of the most striking positive functional associations with sunlight intensity as an example (correlation coefficient 0.89), the deoxyribodipyrimidine photolyase gene (COG0415), the product of which repairs DNA damage caused by exposure to ultraviolet light [40]. Figure 2B shows the abundance of this gene in each of the 24 datasets. It is overwhelmingly abundant in microbial communities that reside at all depth levels that clustered as photic in fig. 1A, whereas the gene is rarely found in those that reside in aphotic zones. Again, Iquique is an exception. Here, although the data from all four depths clustered with the aphotic group of datasets (fig. 1A), the abundance of the photolyase gene at 50 m and 85 m is nevertheless relatively high, while in the 110 m and 200 m datasets this drops to values comparable to those seen in aphotic zones (fig. 2B). Since the 50 m and 85 m levels at Iquique are most probably part of the mixed layer, microorganisms are presumably shuttled to the surface, where they are exposed to sunlight. Therefore, members of these communities are subjected to selective pressure to acquire and/or maintain biological functions that are crucial for survival in a sunlit environment. So, although its PAR value (fig. 1B) indicates that the 50 m layer at Iquique is already aphotic, the relative abundance of photic related COGs (fig. 2A) including photolyase (fig. 2B) argues that it belongs to a facultatively photic zone.

The data for the Sea of Marmara come from a depth of 1 km, which is unquestionably within the aphotic zone. The microbial community at this site was therefore expected to have low levels of the photolyase gene, comparable to those found in other aphotic zones. Paradoxically, however, the deep Marmara sample shows a relatively elevated abundance of this UV-protective gene (fig. 2B). Moreover, the ratio of aphotic to photic related COG abundances for the sample from the Sea of Marmara was found to be only 1.5, similar to that for the 50 m level in the Iquique column and much lower than the mean value for the aphotic datasets of the reference columns (fig. 2A). One possible explanation for this observation is provided by the hydrography and unusual circulation patterns in the Sea of Marmara [41], which receives an inflow of salty (around 3.9%) water from the Mediterranean Sea through the 65 m deep Strait of the Dardanelles. Owing to its density, this mainly euphotic water sinks to the bottom of the Marmara Basin [41]. Therefore, part of the microbial community in the 1-km deep aphotic Marmara environment is actually derived from the surface euphotic zone of the Mediterranean, and is continuously replenished. This would account for the unexpected presence of significant levels of photic genes at this site.

### Effect of oxygen concentration on gene-content in marine microbial communities

**Figure 3:**
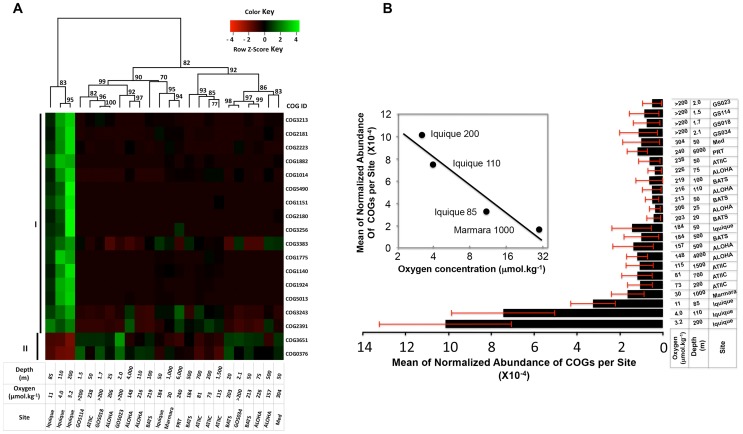
Effect of oxygen concentration on the functional genomic content of microbial communities represented in the 24 datasets. (A) Hierarchical clustering of COGs that significantly influenced by extreme oxygen limitation. Datasets analysis are based on the abundance profile of 18 COGs which significantly differed in abundance levels between an extremely hypoxic environment (Iquique 85-, 110 and 200 m deep, permanent OMZ) and environments with higher D.O. (Fisher's exact test, FDR-corrected, p≤0.05). Bootstrap confidence values for the nodes are shown. Heatmap coloring reflects the Z score of normalized abundances of each COG across all clustered datasets. Roman numbers on the left side of the figure present different groups of COGs as determined by abundance profile across the clustered datasets. (B) Mean abundances of the COGs in group I (figure 3A) as a function of the oxygen concentration. Inset shows the gradual increase in the abundance of these genes in datasets from Marmara 1000 m, Iquique 85 m, Iquique 110 m, and Iquique 200 m, as oxygen concentration drops from 30 µmol.kg^−1^ to 3.2 µmol.kg^−1^.

Marine oxygen minimum zones (OMZs) occur naturally as a result of high oxygen consumption by heterotrophic microorganisms in nutrient-rich zones [35] like the upwelling system of the Humboldt Current (HC). They may also be induced by human activities such as fertilizer run-off and have a great environmental impact by creating zones dominated by microbial communities adapted to hypoxic conditions [42]. Although OMZs have important effects on the diversity, metabolism and physiology of microorganisms [43], [44], [45], the threshold oxygen concentration at which these begin to affect the content of microbial genomes in the ocean is not well defined. In order to clarify this issue, the three deepest Iquique datasets (85, 110 and 200 m) were chosen as reference hypoxic datasets because they represent the persistent OMZ present at this site [36] and comprise the datasets with the lowest oxygen concentrations among the 24 datasets studied in this work ([O_2_] = 11.0, 4.0 and 3.2 µmol.kg^−1^ at 85, 110 and 200 m, respectively). To avoid interference from any effects related to sunlight incidence, we removed members of the photic/aphotic global-core depth-related COGs from the list of 162 differentially abundant COGs that are statistically significant for the Iquique column. The normalized abundance medians of the remaining 149 COGs from these hypoxic Iquique samples were then compared with the corresponding values for the other 21 datasets (for details see Methods). As shown in fig. 3A (table S5 in file S1), 18 COGs in all were found to be significantly influenced by extreme oxygen limitation (p< = 0.05, FDR-corrected); 16 of these clustered in group I, and two in group II. Interestingly, approximately 43% of the COGs that are significantly more abundant in extremely hypoxic samples (Group I in fig. 3A) are related to nitrate metabolism.

Since the oxygen tension in the OMZ at the Iquique site is very low, the ability to use alternate final electron acceptors for the replenishment of oxidative potential and energy production coupling becomes imperative for survival. This probably accounts for the fact that a number of the COGs overrepresented in this zone (COG2223, COG5013, COG1140, COG2181, COG2180, COG3256 and possibly COG3213) are related to nitrate reduction. The abundance of an enzyme involved in fermentation and specifically inactivated by oxygen (COG1882) was also significantly high. In addition, enzymes that are implicated in methanogenesis (COG1151) and pyruvate metabolism (COG1014) exhibited increased abundance in these datasets. Conversely, genes whose products require molecular oxygen for their activities - such as catalase (COG0376, fig. 3A, table S5 in file S1) - are poorly represented in this extremely hypoxic environment.

Analysis of the mean abundances of the COGs in group I as a function of the oxygen concentration reveals that these COGs are distinctively and overwhelmingly abundant in microbial communities from samples in which oxygen concentration was ≤11 µmol.kg^−1^ (fig. 3B). It is intriguing to note the gradual increase in the abundance of these genes in datasets as oxygen concentration drops from 30 µmol.kg^−1^ to 3.2 µmol.kg^−1^ (inset fig. 3B).

The fact that the relative abundances of genes in group I increases dramatically at oxygen concentrations of 11 µmol.kg^−1^ or less suggests that this level of oxygen represents the threshold at which selective effects on genome content in favor of anaerobic lifestyles set in.

Based on the content of the core sets of photic/aphotic depth-related COGs from all sites presented in fig. S3A, it appears that microbial communities living in photic zones share the same functions, regardless of site location. Thus, with respect to this zone, we do not see any specific branches clustering together that can be attributed to environmental conditions that are unique to any particular geographical location. The aphotic samples (based on PAR values), however, do display distinct branching profiles of genomic content that can be correlated with specific environmental conditions and oxygen concentrations. The three mixed-water layers, Iquique 50 and 85 m and Marmara 1 km (with aphotic:photic ratios of between 1.3 to 1.8; refer to fig. 2A), in which limitation of light and oxygen in these aphotic zones exert selective pressures, cluster together (fig. S3A, see also fig. S3B branch I), while the extremely hypoxic samples in the aphotic zone at Iquique (110 and 200 m) formed a separate cluster on the other side of the tree (fig. S3A, see also S3B branch II). The abundances of two COGs, photolyase (COG0415) and nitrate reductase (Alpha Subunit; COG5013), taken as representative of core photic and aphotic related COGs that contributed to the functional branching in these aphotic zone, are presented in fig. S3B. The results again argue that the concentration of oxygen at which selective pressure begins detectably to favor the acquisition of adaptations for survival under hypoxic conditions lies below that of the Iquique 85 m sample (11 µmol.kg^−1^), which is assigned to branch I.

### Taxonomical distribution of photic and aphotic depth-related COGs

**Figure 4:**
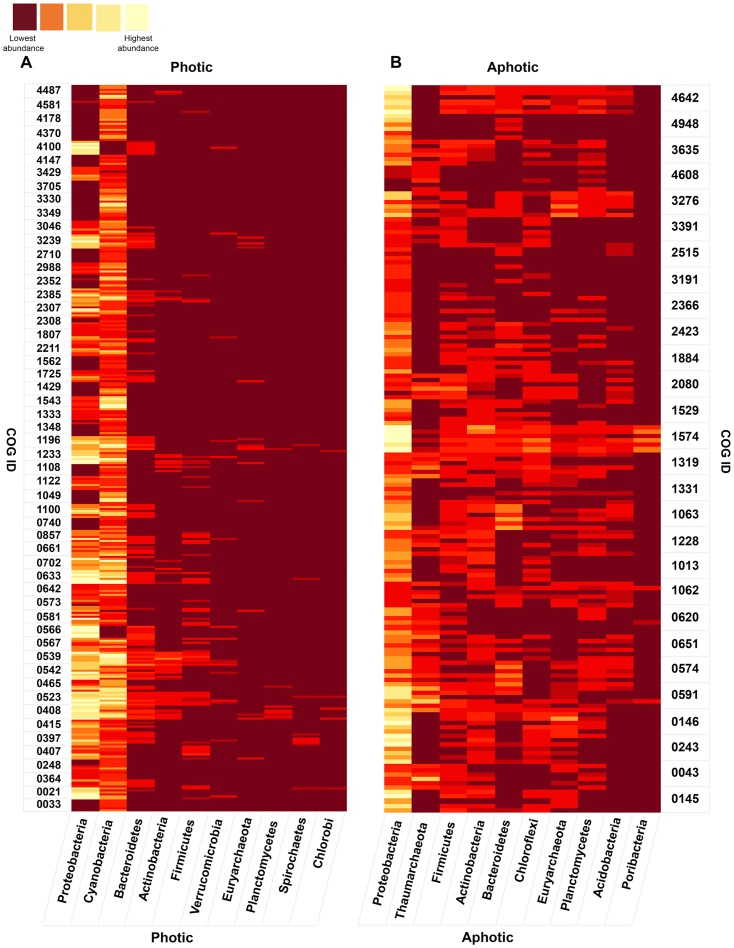
Taxonomical assignments of photic and aphotic depth-related COGs at the phylum level for the selected datasets. Reads assigned to COGs were compared to the NCBI nr database using BLASTX (E-value cutoff 1e-05) and the taxonomical identifiers of the best matches were retrieved. Subsequently, tables of relative frequency of taxa per COG per dataset were generated (tables S6A and S6B in file S1) and presented as heatmaps. The 10 phyla that have the highest average numbers of photic (**A**) and aphotic (**B**) COGs per site are presented. The taxa are sorted by the decreasing of their abundance. Datasets of the shallowest samples of the ALOHA, ATII and BATS water columns and single-depth datasets from GOS018, GOS023, GOS034, GOS114 and Mediterranean sites were used for the analysis of the photic related COGs, whereas the deepest datasets from the ALOHA, ATII and BATS water columns, and single-depth datasets from Marmara, PRT and ALOHA (4,000 m), were employed in the case of the aphotic related COGs. This analysis was based on a total of 36,938 sequences (24919 sequences are photic, and 12019 are aphotic).

The establishment of collections of core photic and core aphotic depth-related COGs raises the question of how such functions are disseminated among phylogenetic clades present in the different environments covered by our analysis. To address this point, we generated taxonomic assignments, at the phylum level, for each core photic and aphotic depth-related COG based on the taxonomic identifiers of the respective reads (for details see Materials and Methods, and tables S6A and S6B in file S1). The results show that sequences related to core photic COGs derive from far fewer taxonomic clades than those of core aphotic ones. This reflects the predominance of certain taxa in most euphotic environments and the higher diversity of taxa found in deeper oceanic environments (fig. 4, and tables S6A and S6B in file S1). Moreover, the dominant taxon for any given COG is, with few exceptions, shared among all the sites analyzed.

The taxonomic distribution of the 54 core photic related COGs at the phylum level (fig. 4A, table S6A in file S1) reveals that the vast majority (91%) of them are most probably contributed by Proteobacteria (46%) and Cyanobacteria (45%). The taxonomic distribution of the 28 core aphotic related COGs at the phylum level is much more diverse than that observed for the photic functions. Although Proteobacteria are again predominant (50%), a total of ten phyla are represented at levels greater than 1%, compared to only five for the photic set (fig. 4B, table S6B in file S1).

The increased diversity of microbial communities in aphotic environments is perhaps the most striking result of the phylogenetic analysis of the core COGs. It can be attributed to adaptation to the wider variety (and lower abundance) of exploitable resources available in the aphotic zone, which contrasts with overwhelming importance of photosynthetic energy production in the photic zone. This interpretation is supported by the biochemical functions assigned to core photic/aphotic related COGs observed in our work.

In this study, using metagenomics-based functional profiling analysis, we have defined core sets of photic and aphotic depth-related biological activities that are shared by different sites distributed around the global oceans. These two sets of depth-related functional biological activities were used to provide insight into the interplay between light intensity - the most significant abiotic evolutionary force in the oceans - and the genetic and ecological differentiation of marine microbial communities. In addition, in conjunction with a set of functional biological activities related to extreme hypoxia, we provide a comprehensive picture of microbial biological activities in oceanographic settings in which light and oxygen imprint its effect in nominally aphotic zones. Moreover, our work highlights the importance of developing reference sets of functional biological activities that can be used as a tool for the diagnosis of the physiological and biochemical capabilities, and phylogenetic profiling, of marine microorganisms. This approach should be particularly helpful for investigations of the impact of anthropogenically induced environmental changes on marine ecosystems.

## Supporting Information

Figure S1 **Locations of the 11 sites and numbers of sequenced reads in the 24 datasets.**
(TIFF)

Figure S2 **Hierarchical clustering of datasets from the three reference water columns (ATII, ALOHA, BATS).** This analysis is based on the normalized abundance profile of 176 selected COGs that significantly differed in abundance within at least one of column (Fisher's exact test, FDR-corrected, p ≤ 0.01) (table S2 in file S1). Heatmap coloring reflects the Z score of normalized abundances of each COG across clustered datasets. Roman numbers on the left side of the figure present different groups of COGs as determined by abundance profile across the clustered datasets.
(TIFF)

Figure S3 **Hierarchical clustering of the 176 depth-related COGs in the 24 datasets.** **(A)** Heatmap of the datasets from 11 diverse marine sites (24 datasets). Refer to legend of figure 1A for details. Roman numbers on the left side of the figure present different groups of COGs as determined by abundance profile across the clustered datasets. The dendrogram of this figure is also presented in fig. 1A. (B) Distinct branching profiles of genomic content of the aphotic samples. The aphotic branch in figure S3A is presented together with the level of oxygen in each site and the normalized abundances of Deoxyribodipyrimidine photolyase (COG0415), and Nitrate reductase (Alpha Subunit; COG5013). The values of the normalized abundance of both COGs were obtained from table S4A and S5 in file S1.
(TIFF)

File S1 **Table S1, Sites locations, datasets, sampling and sequencing methods. Table S2, List of 176 depth-related COGs shared by the three reference columns, ATIIC, ALOHA and BATS.** Fisher's exact test (p ≤ 0.01, FDR corrected) was employed to identify COGs that presented a statistically significant difference in abundance between at least two depths of a reference column. COG cluster roman numbers refers to groups of COGs displaying similar normalized abundance profile. COGs are ordered as in figure S2. **Table S3, List of 176 depth-related COG set in all 24 datasets.** Fisher's exact test (p ≤ 0.01, FDR corrected) was employed to identify the 176 depth-related COG set (table S2 in file S1) in all 24 datasets. Among these, the ones that were present in at least one dataset of each reference column were selected for subsequent comparative analysis of all datasets. COG cluster roman numbers refers to groups of COGs displaying similar abundance profile in the hierarchical clustering of all 24 datasets. COGs are ordered as in figures 1A and S3A. **Table S4, A. List of 54 photic global-core, depth-related COGs.** Welch's test (p ≤ 1E-04, FDR corrected) was employed to identify COGs that presented a statistically significant difference in the mean abundance between the photic and the aphotic group of datasets (see details in Methods). Photic related COGs are presented in the same order as shown in group I Fig. 1C. **B. List of 28 aphotic global-core depth-related COGs.** Welch's test (p ≤ 1E-04, FDR corrected) was employed to identify COGs that presented a statistically significant difference in the mean abundance between the photic and the aphotic group of datasets (see details in Methods). Aphotic related COGs are presented in the same order as shown in group II Fig. 1C. **Table S5, List of 18 Oxygen Minimum Zone-related COGs.** Wilcoxon test (p ≤ 0.05, FDR corrected) was employed to identify COGs that significantly differed in abundance between the highly hypoxic group of datasets (Iquique 85-, 110- and 200-m deep samples) and the group of remaining datasets with higher oxygen concentration (details in Methods). COGs are presented in the same order as that in the heatmap (fig. 3A).**Table S6. A. Taxonomic distribution of the photic COGs at the phylum level.** Phylogenetic analysis was performed based on the NCBI taxonomic identifiers of the best matches to the nr database. For each COG, the rawcounts of assigned taxa is presented for eight different photic datasets: the shallowest samples of the reference columns, and five singledepth samples (details in Methods). The 10 phyla that have the highest average numbers of photic COGs per site are presented in fig. 4A. **B. Taxonomic distribution of the aphotic COGs at the phylum level.** Phylogenetic analysis was performed based on the NCBI taxonomic identifiers of the best matches to the nr database. For each COG, the rawcounts of assigned taxa is presented for six different aphotic datasets: the deepest samples of the reference columns and three single-depth samples. The 10 phyla that have the highest average numbers of aphotic COGs per site are presented in Figure 4B.
(XLS)

Methods S1 **Sample collection, DNA isolation, pyrosequencing, and data processing.**
(DOC)
