# Supplementary figures and images for: Core Microbial Functional Activities in Ocean Environments Revealed by Global Metagenomic Profiling Analyses
Source: PLoS One. 2014 Jun 12;9(6):e97338. doi: 10.1371/journal.pone.0097338 (PMC4055538; doi:10.1371/journal.pone.0097338)

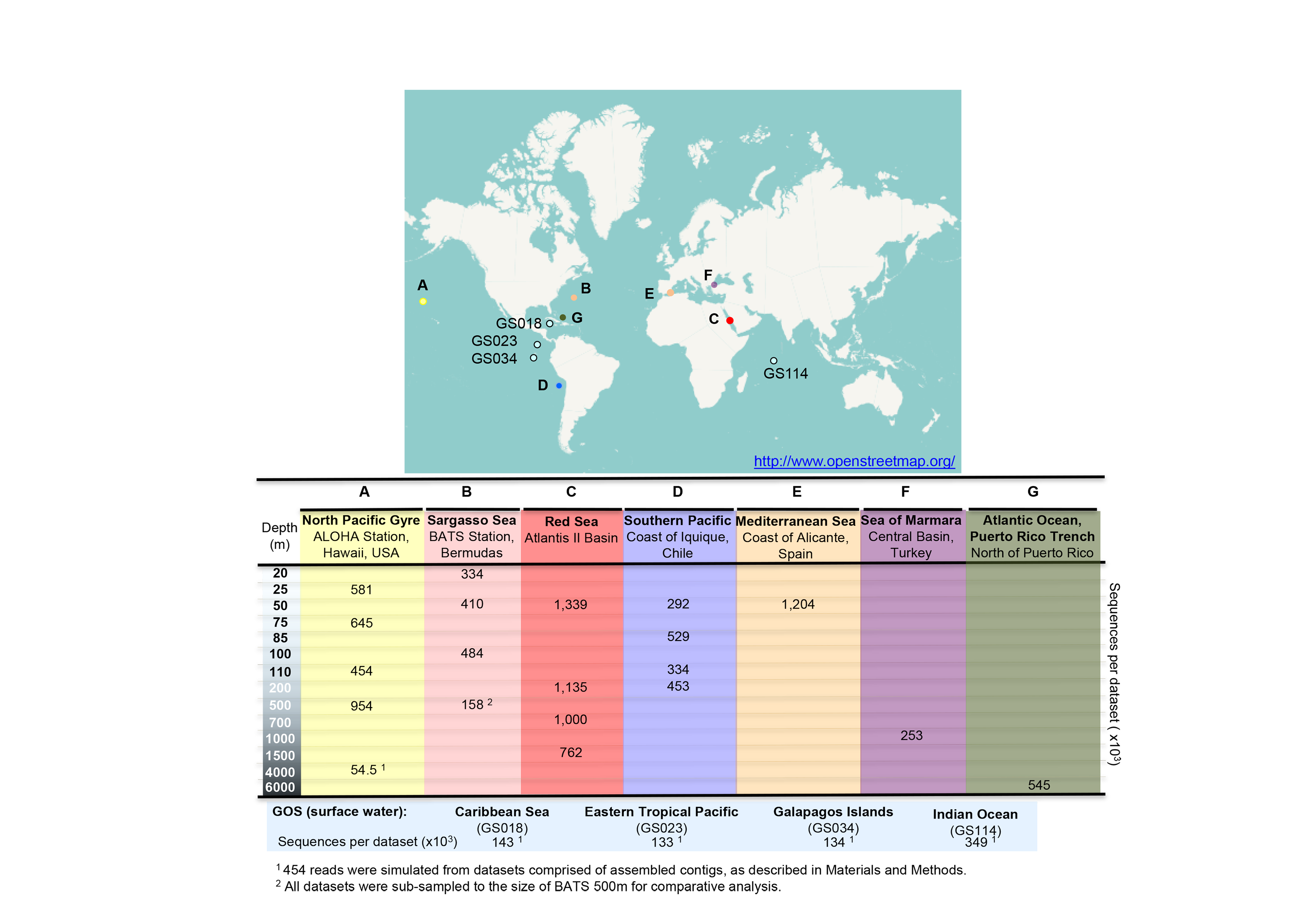

Supplement: Figure S1 — Locations of the 11 sites and numbers of sequenced reads in the 24 datasets. (TIFF) [file pone.0097338.s001.tif]

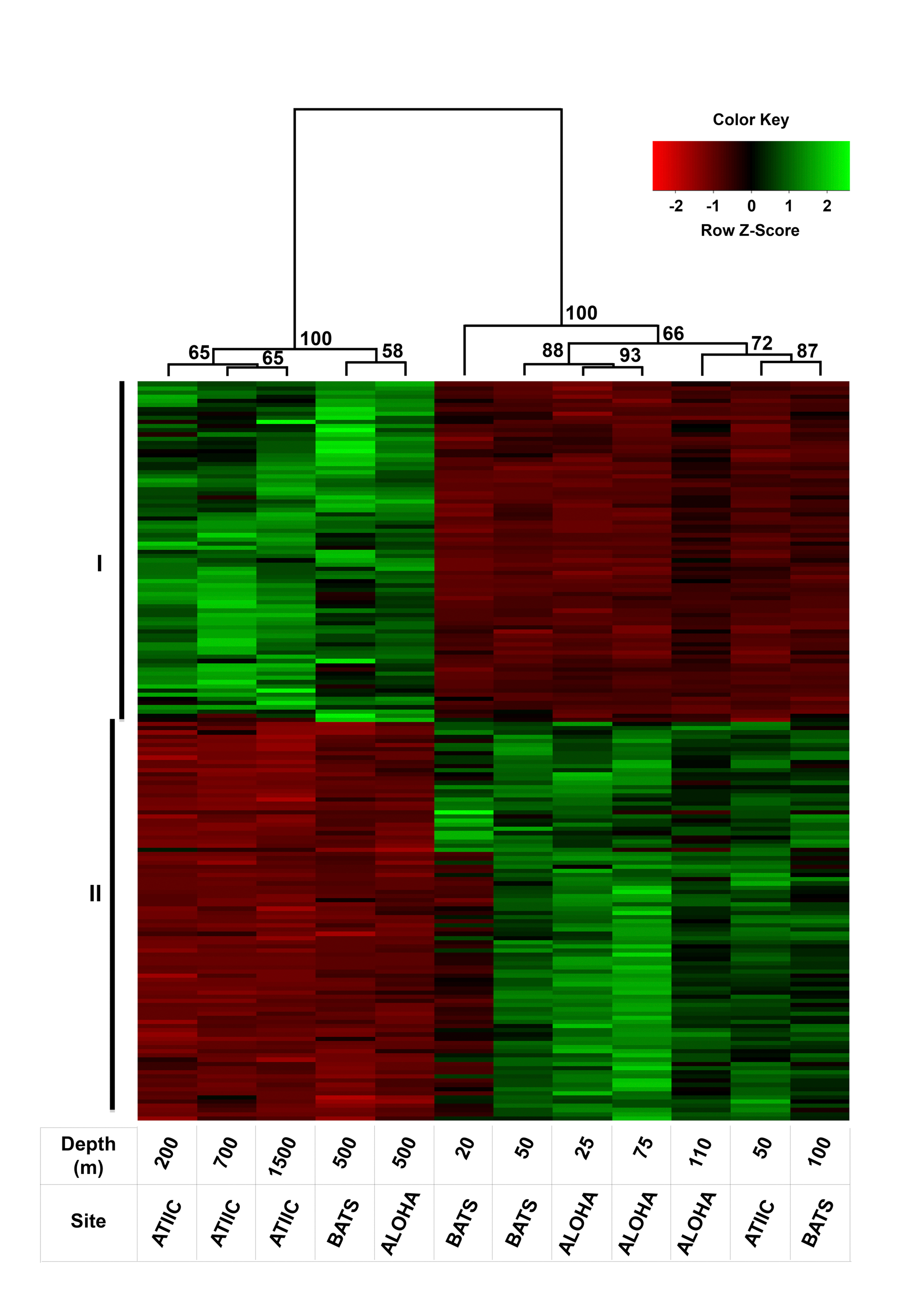

Supplement: Figure S2 — Hierarchical clustering of datasets from the three reference water columns (ATII, ALOHA, BATS). This analysis is based on the normalized abundance profile of 176 selected COGs that significantly differed in abundance within at least one of column (Fisher's exact test, FDR-corrected, p ≤ 0.01) (table S2 in file S1). Heatmap coloring reflects the Z score of normalized abundances of each COG across clustered datasets. Roman numbers on the left side of the figure present different groups of COGs as determined by abundance profile across the clustered datasets. (TIFF) [file pone.0097338.s002.tif]

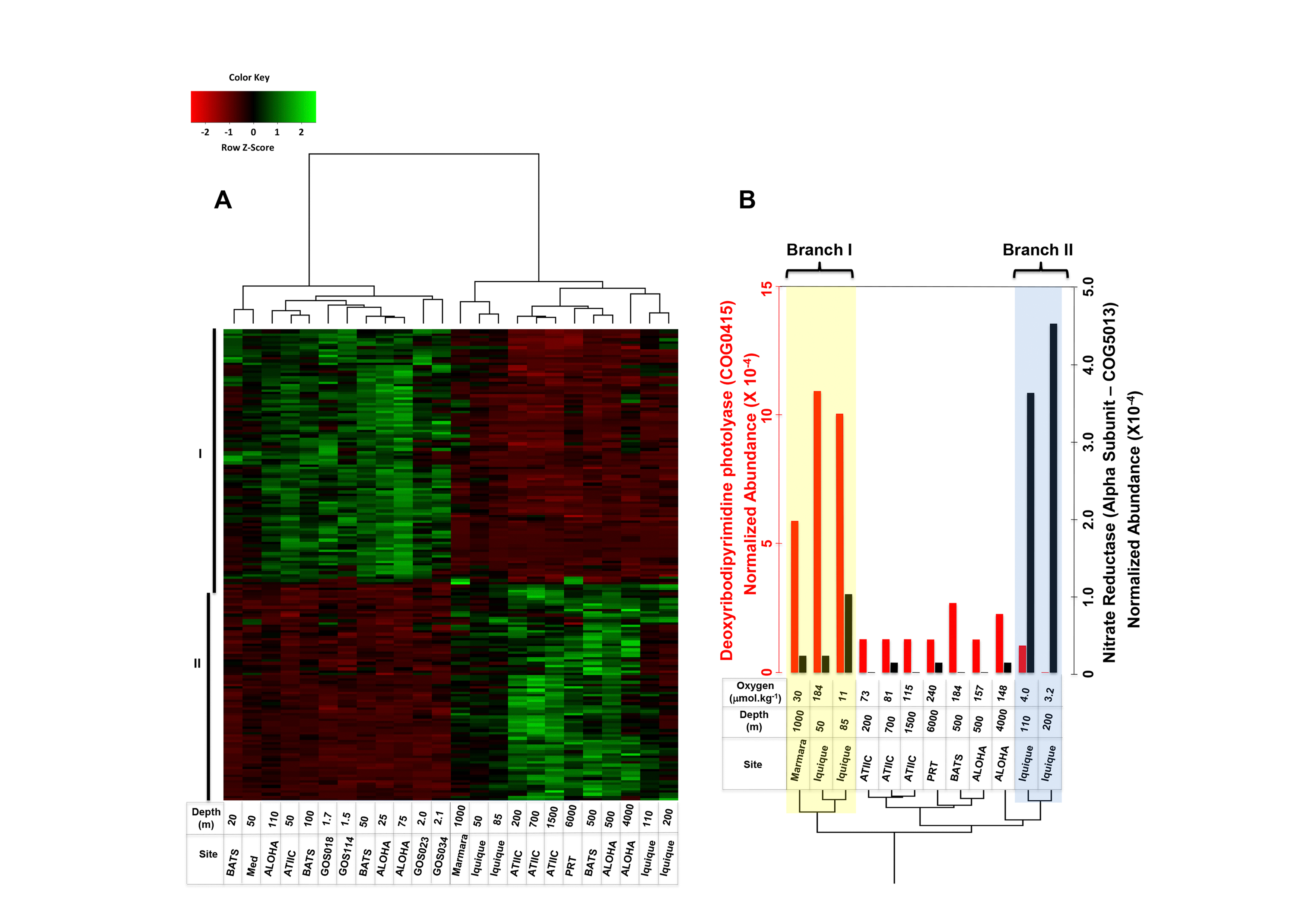

Supplement: Figure S3 — Hierarchical clustering of the 176 depth-related COGs in the 24 datasets. (A) Heatmap of the datasets from 11 diverse marine sites (24 datasets). Refer to legend of figure 1A for details. Roman numbers on the left side of the figure present different groups of COGs as determined by abundance profile across the clustered datasets. The dendrogram of this figure is also presented in fig. 1A. (B) Distinct branching profiles of genomic content of the aphotic samples. The aphotic branch in figure S3A is presented together with the level of oxygen in each site and the normalized abundances of Deoxyribodipyrimidine photolyase (COG0415), and Nitrate reductase (Alpha Subunit; COG5013). The values of the normalized abundance of both COGs were obtained from table S4A and S5 in file S1. (TIFF) [file pone.0097338.s003.tif]
