# Supplementary material for: Core Microbial Functional Activities in Ocean Environments Revealed by Global Metagenomic Profiling Analyses
Source: PLoS One. 2014 Jun 12;9(6):e97338. doi: 10.1371/journal.pone.0097338 (PMC4055538; doi:10.1371/journal.pone.0097338)
Supplement: Methods S1 — Sample collection, DNA isolation, pyrosequencing, and data processing. (DOC) [file pone.0097338.s005.doc]

**Methods S1**

**Sample collection, DNA isolation, pyrosequencing, and data processing**

*Sample collection, DNA isolation, and pyrosequencing.* Ten Niskin bottles mounted on a CTD rosette were used to collect water samples from four different depths, 50 m, 200 m, 700 m, and 1,500 m. On average, 120 liters of each depth sample was sequentially filtered through mixed-cellulose-ester filters with pore sizes of 3.0, 0.8 and 0.1 µm respectively, using Millipore 293-mm stainless steel sanitary filter holders (Durapore, Millipore, Billerica, MA, USA). The filters were then placed in sucrose lysis buffer [1] at -20^o^C for delivery to the Genomics Facility at AUC in Cairo, where the samples were stored in a -70^o^C freezer until further processing for DNA extraction.

Environmental DNA was isolated from each 0.1-µm filter as described by Rusch and colleagues [1], incorporating the modification in the CTAB treatment detailed in the DOE-Joint Genome Institute protocol (http://my.jgi.doe.gov/general/; DNA Isolation Bacterial CTAB Protocol). Measurement of DNA concentration was performed using a NanoDrop3300 Fluorospectrometer (Thermo Scientific, USA) and the Quant-iT™ PicoGreen® dsDNA kit (Invitrogen, USA).

Environmental DNA isolated from microbes (in the 0.1 to 0.8 µm size range) collected on 0.1-µm filters were used to construct libraries as recommended by GS FLX Titanium library guide (Roche, Germany). Sequencing was performed on a GS FLX pyrosequencer using the Titanium pyrosequencing kit (454 Life Sciences).

The sequences obtained for each library were subjected to standard processing by the Roche® Genome Sequencer Data Analysis Software package (GS) version 2.0.00.22, using the default parameters to obtain standard flowgram, sequence quality and fasta files devoid of adapter sequences and low-quality regions. As a final check on quality, we removed ghost sequences [2] from our datasets using cdhit-454 [3] with alignment identity ≥ 98%.

*Publically available datasets, 454 shotgun read simulation, sub-sampling, and functional assignment using the eggNOG database.* Fasta files of the four GOS [1,4] and the Mediterranean [5] datasets were downloaded from MG-RAST (http://metagenomics.anl.gov/v2/) [6], while the files corresponding to Marmara [7], PRT [8], ALOHA and BATS [9], Iquique [10] datasets were obtained from the NCBI Sequence Read Archive (SRA, http://trace.ncbi.nlm.nih.gov/Traces/sra) [11]. The ALOHA4000 dataset [12] was obtained from the NCBI WGS sequencing project database (http://www.ncbi.nlm.nih.gov/ Traces/wgs).

Each of the datasets comprising assembled Sanger sequenced reads (GOS and the ALOHA 4000) served as a source of linear contig templates that was used to simulate a corresponding dataset made up of pyrosequenced reads with the aid of MetaSim [13]. Simulation parameters were set to 200 cycles per error model, generating unpaired reads with 400 bp mean length. A total of 614,591 sequences (average number of reads for the 454 shotgun datasets) were generated. Sub-sampling of the 24 datasets to a common sequencing depth was performed using a custom Perl script. The sub-sampling size chosen was equal to the total size of the smallest dataset (BATS 500 m, 158,407 reads).

To perform sequence similarity searches against the public databases, open reading frames (ORFs) were predicted from datasets that were assembled (i.e., GOS and PRT datasets) or obtained by Sanger sequencing (ALOHA4000) using MetaGene software [14]. Sequences from datasets composed of unassembled 454 reads (ATIIC, ALOHA, BATS and Iquique water columns) were subjected to BLASTX [15] similarity search against the eggNOG v2.0 database [16]. We deployed mpiBLAST, a parallel version of commonly used BLAST on IBM BLUEGENE P at King Abdullah University of Science and Technology (KAUST) using a maximum e-value threshold of 1e-05. Results were parsed to include only NCBI COGs that were mapped to only one protein in the database. Sequences assigned to more than one COG were discarded from further analyses. Raw COG abundance measures were converted to a fraction representing the relative contribution of each COG count to the total number of sequences assigned to COGs for each dataset to account for different levels of sampling across multiple datasets [12] [17]. Normalized abundances of the COGs were determined based on the total number of sequences assigned to COGs for each dataset.

*Analysis of differentially abundant COGs.* Hierarchical clustering of metagenomic datasets was performed based on normalized COGs abundance measures. Datasets clustering together were identified as well as differentially abundant COGs across different groups of datasets. Further, COGs that differ significantly in abundance between at least two datasets were identified using Fisher’s exact test. COGs whose p values exceed a specified threshold (e.g. p ≤ 0.05) following a false discovery rate correction for multiple comparisons were inferred to be differentially abundant across the two datasets/groups under analysis. Statistical tests, hierarchical clustering and heatmaps generation were computed/generated using the free software environment for statistical computing and graphics R version 2.11.1 (www.r-project.org).

*Hierarchical clustering.* Hierarchical clustering of metagenomic datasets and calculation of approximately unbiased p-values by multiscale bootstrap resampling [18] were performed using the pvclust package (v. 1.2-2, Suzuki, R. & Shimodaira, H., developed for the R platform, http://www.is.titech.ac.jp/~shimo/prog/pvclust/). In order to generate distance matrices for dataset clustering, complete linkage and (1-Spearman’s correlation) metrics were applied to both columns and rows of tables containing COG normalized abundance data.

**References**

1. Rusch DB, Halpern AL, Sutton G, Heidelberg KB, Williamson S, et al. (2007) The Sorcerer II Global Ocean Sampling expedition: northwest Atlantic through eastern tropical Pacific. PLoS Biol 5: e77.

2. Gomez-Alvarez V, Teal TK, Schmidt TM (2009) Systematic artifacts in metagenomes from complex microbial communities. ISME J 3: 1314-1317.

3. Niu B, Fu L, Sun S, Li W (2010) Artificial and natural duplicates in pyrosequencing reads of metagenomic data. BMC Bioinformatics 11: 187.

4. Yooseph S, Nealson KH, Rusch DB, McCrow JP, Dupont CL, et al. (2010) Genomic and functional adaptation in surface ocean planktonic prokaryotes. Nature 468: 60-66.

5. Ghai R, Martin-Cuadrado AB, Molto AG, Heredia IG, Cabrera R, et al. (2010) Metagenome of the Mediterranean deep chlorophyll maximum studied by direct and fosmid library 454 pyrosequencing. ISME J 4: 1154-1166.

6. Meyer F, Paarmann D, D'Souza M, Olson R, Glass EM, et al. (2008) The metagenomics RAST server - a public resource for the automatic phylogenetic and functional analysis of metagenomes. BMC Bioinformatics 9: 386.

7. Quaiser A, Zivanovic Y, Moreira D, Lopez-Garcia P (2011) Comparative metagenomics of bathypelagic plankton and bottom sediment from the Sea of Marmara. ISME J 5: 285-304.

8. Eloe EA, Fadrosh DW, Novotny M, Zeigler Allen L, Kim M, et al. (2011) Going deeper: metagenome of a hadopelagic microbial community. PLoS One 6: e20388.

9. Martinez A, Tyson GW, Delong EF (2010) Widespread known and novel phosphonate utilization pathways in marine bacteria revealed by functional screening and metagenomic analyses. Environ Microbiol 12: 222-238.

10. Stewart FJ, Ulloa O, DeLong EF (2012) Microbial metatranscriptomics in a permanent marine oxygen minimum zone. Environ Microbiol 14: 23-40.

11. Wheeler DL, Barrett T, Benson DA, Bryant SH, Canese K, et al. (2008) Database resources of the National Center for Biotechnology Information. Nucleic Acids Res 36: D13-21.

12. Konstantinidis KT, Braff J, Karl DM, DeLong EF (2009) Comparative metagenomic analysis of a microbial community residing at a depth of 4,000 meters at station ALOHA in the North Pacific subtropical gyre. Appl Environ Microbiol 75: 5345-5355.

13. Richter DC, Ott F, Auch AF, Schmid R, Huson DH (2008) MetaSim: a sequencing simulator for genomics and metagenomics. PLoS One 3: e3373.

14. Noguchi H, Park J, Takagi T (2006) MetaGene: prokaryotic gene finding from environmental genome shotgun sequences. Nucleic Acids Res 34: 5623-5630.

15. Altschul SF, Madden TL, Schaffer AA, Zhang J, Zhang Z, et al. (1997) Gapped BLAST and PSI-BLAST: a new generation of protein database search programs. Nucleic Acids Res 25: 3389-3402.

16. Muller J, Szklarczyk D, Julien P, Letunic I, Roth A, et al. (2010) eggNOG v2.0: extending the evolutionary genealogy of genes with enhanced non-supervised orthologous groups, species and functional annotations. Nucleic Acids Res 38: D190-195.

17. White JR, Nagarajan N, Pop M (2009) Statistical methods for detecting differentially abundant features in clinical metagenomic samples. PLoS Comput Biol 5: e1000352.

18. Shimodaira H (2004) Approximately unbiased tests of regions using multistep-multiscale bootstrap resampling. Annals of Statistics 32: 2616-2641.
